# Quacks

**Published:** 1908-10

**Authors:** M. A. Clark

**Affiliations:** Macon, Ga.


### Journal-Record of Medicine

Successor to Atlanta Medical and Surgical Journal, Established 1855, and Southern Medical Record, Established 1870.

OWNED BY THE ATLANTA MEDICAL JOURNAL CO.

Published Monthly.

*Official Organ Fulton County Medical Society, State Examining Board, Presbyterian Hospital, Etc.*

EDGAR G. BALLENGER, M. D., Editor.
BERNARD WOLFF, M. D., Supervising Editor.
A. W. STIRLING, M. D., C. M., D. P. H., J. S. HURT, B. Ph., M. D., Associate Editors
E. W. ALLEN, Business Manager.

COLLABORATORS
DR. W. F. WESTMORLAND, General Surgery.
F. W. McRAE, M. D., Abdominal Surgery.
H. F. HARRIS, M. D., Pathology and Bacteriology.
E. B. BLOCK, M. D., Diseases of the Nervous System.
MICHEL HOKE, M. D., Orthopedic Surgery.
CYRUS W. STRICKLER, M. D., Legal Medicine and Medical Legislation.
E. C. DAVIS, A. B., M. D., Obstetrics.
E. G. JONES, A. B., M. D., Gynecology.
R. T. DORSEY, Jr., B. S. M. D., Medicine.
L. M. GAINES, A. B., M. D., Internal Medicine.
J. N. LeCONTE, M. D., Diseases of the Stomach and Intestines
L. B. CLARKE, M. D., Pediatrics.
EDGAR PAULIN, M. D., Opsonic Medicine.
R. R. DALY, M. D., Medical Society.
A. W. STIRLING, M. D., Etc., Diseases of the Eye, Ear, Nose and Throat.
BERNARD WOLFF, M. D., Diseases of the Skin.
E. G. BALLENGER, M. D., Diseases of the Genito Urinary Organs.

VOL. X. OCTOBER, 1908. No. 7

### QUACKS.

BY M. A. CLARK, M. D., MACON, GA.

"With monstrous promise they delude the mind,
And thrive on all that tortures human kind."

It is impossible to ascertain with any degree of exactness when quacks first began to prey upon the sufferings of mankind. We find mention of them contemporaneous with Aesculapius, the god of medicine; and we have abundant evidence of their existence at the present day.

Words are hardly adequate to portray to you the wonderful progress made by man since the time of Aesculapius. Civilization has made marvelous strides. The whole world has been revolutionized by education; and every branch of science has kept pace with this advancement. When we consider what our profession was then and what it is now, we are amazed at the progress made, and we marvel that there is still a place for the charlatan in medicine.

The eighteenth century has been called the "Quack Century." I apprehend that were the same author living in this the brilliant dawn of the twentieth century, he would not hesitate to predict that this century will out quack the "Quack Century."

Judging from the etymology of the word, the first quacks were those who talked extravagantly of the cures they had made and boasted of the usual skill they pretended to possess. You may easily find such as these thriving in this day and generation.

Then came the quacks who advertised their cures and made boast of their skill in the newspapers, and on cards and circulars printed in bold type and handed out to every passer by.

Steele in his Spectator of July, 1712, says: "Quack doctors, who publish their great abilities in little green billets, distributed to all who pass by, are to a man impostors and murderers." I doubt not but that the editors of our great newspapers of today would voice the same sentiment, and yet we find these journals full of advertisements of quacks and quack medicines.

We, too, have the advertising quack, and he differs but little from those of Steele's day, except that he is bolder and makes more frequent use of printers' ink, to delude the minds of the public. This is really the least dangerous of all quacks, because the methods are so bold and the motives so apparent that not a few recognize and avoid them.

During the life of Aesculapius there were those who stole his teachings and, under the guise of some queer name or party, preyed upon the ills of the credulous people. Hippocrates was beset by the same class of quacks. They have prospered throughout the succeeding ages and are today quite numerous though unedr new names. They steal what meager knowledge of medicine they have from the Regular Profession, yet spend much time and energy in encouraging the prejudices of the laity and ridiculing the real Science of Medicine. I am sure you all readily recall some under a new pathy, others under a so-called new religion, and not a few of us know how well they deceive the public, and how the public seem to revel in this deception.

They are multiplying at a rapid rate and are growing bolder and bolder in their practices. They set themselves up as experts more learned than the regular profession and even demand that they be put on the same legal footing with us. Notwithstanding all this, they are not so dangerous to the people nor so injurious to our profession as another class of quacks, whom I now ask you to consider for a few moments.

There are certain little events in the childhood of all of us that are small indeed as compared with the realities of life and yet impress themselves more indelibily upon us than those of far greater importance. Are there any among us who will ever forget the first time we ever went to a circus or took a chew of tobacco? The very mention of these things carry us back to those happy days and we are living them over again or wishing that they could come again.

A few incidents of a medical character linger vividly around the memory of my childhood and seem as real as if it were but yesterday that they occurred. One, the veritable night mare of my childhood, is the old cup with a piece broken out and dark from the use of years, the bottle of castor oil, the old fashioned fire place with the little pile of hot ashes raked out to heat the cup that the oil might pour more readily, the cup of coffee to take afterwards to take the taste out," and last but by no means least, the peach tree switch so necessary to arouse my courage to the point of swallowing and nauseous dose. Can I ever forget that scene?

You may flavor, disguise, castor oil if you will,
But belch, and the taste will linger with you still.

With due apologies to the poet.

Another memorable epoch of childhood days is the event of the family physician when some of us were sick. Well do I remember how he sat by the cradle of my little sick brother and entertained himself with telling us of the many sick cases just like that only much worse, and how he was called just in time to save them from an awful death by his magic skill. How he came to see me once and found me "threatened with pneumonia" and was able to break it up and save me. Do you wonder that I would sit there agape, taking it all in and wondering how any one could know so much and be always so ready to do just the right thing at the proper time?

Have you ever heard of a medical man standing on the street corner or in the drug store talking in a loud and boastful manner to a crowd of acquaintances about the wonderful operations he has performed; how the poor sufferer was cut all to pieces or his intestines were riddled with bullets and may be the liver had been penetrated, and yet with his great skill and dexterity the operation was quickly done, and the patient made a rapid and wonderful recovery; or of the number of cases of midwifery he has had, all difficult cases, and has never lost a case; or of the hundreds of cases of typhoid fever he has cured? He would love to talk longer with them but is so very busy that he must go.

How often has the poor patient been forced to lie there and listen to her doctor tell of so many cases that he has cured, all of them harrowing to her very soul and making her more nervous and restless. So full of self and so absolutely convinced of his own importance that he forgets the real motive for which he was called, and oft times his patient is worse than before he visited her.

Such is the quack within our own ranks, a graduate of a regular school of medicine, licensed as we are, and exercising all of the privileges belonging to us. He is a frequent boaster of his thorough familiarity with the Code of Ethics about which he really knows nothing, and is ever talking of his observance of Ethics and the absolute ignorance of it as shown by his contemporary. While this can hardly be termed a "brain storm," it is undoubted evidence of an exaggerated ego of a chronic character.

These men rarely have time to attend their medical societies and, too, never hear anything worth listening to if they do attend. Yet they spend much time in boasting of their skill and keeping themselves conspicuously before a gullible public. No real physician of the most perfect health and the greatest powers of endurance is able to do the amount of work and study they claim to do.

All of these men are not wholly worshipers of self and filthy lucre, but some fall into the habit because they have not the courage nor patience to wait to build up on that firm, but true foundation, Real Ability.

Hasten the day we shall be able to say that ours, the greatest and noblest of professions is wholy free from anything that even savors of quackery.

We must not forget to mention the class of quacks, who do not treat nor visit the sick, but who have discovered some panacea for all diseases and spend their time and energies in advertising it to the public so fond of self medicating. Our minds are scarcely able to grasp the enormous amount of money spent each year for these remedies.

We may easily recall a remedy of this class advertised so insidiously that it deceived not only the laity but also many of the profession. Thousands of dollars have been made by them within a short period of time, and some of us have aided them so much in this by prescribing and recommending their preparation. I am sure you will readily call to mind many such remedies.

Now the all absorbing question, why do these quacks continue to live and thrive? The answer was given years ago: "Quackery and the love of being quacked are in human nature as weeds in the fields." It applies with equal force today.

Contemporaneous with the reason for quacks was given the remedy for the removal: "If all understood medicine, there would be none to take his quack medicine."

The laity have advanced in every branch of knowlege save that of Medicine. Only a short time ago, the editor of one of Georgia's best daily papers, in commenting upon doctors, said: "When it comes to doctors, one doctor is as good as another." If a man is called doctor, or peradventure is licensed to practice medicine, he is to the average person as good as the physician who has devoted years to study and research. Even the law recognizes him as much an expert as the most learned in the profession.

Then, Gentlemen, the real remedy for this great evil is to educate the people in such a way that they may be able and willing to discriminate. After they are thus educated, there will be no place nor demand for the quack, and our profession will be reveredand prized as it so well deserves to be.

This education must come from the medical profession and must be wisely and carefully done. We spend much time and great energy and some money in learning a preventive for some dread disease and count all well spent. Can any prophylaxis be worth more to suffering humanity than that which will save them from the ravages of the quack. Like every undertaking by medical men for the good of mankind, this must be accomplished without any aid or much encouragement from the laity.

Our efforts at organization, Brethren, are but the right step in this direction. The more thoroughly organized we become, the more easily can we arouse the profession to greater activity and the more rapidly educate the people to a full appreciation of the real value of our profession.

When this has been accomplished, the weeds of quackery will have been plucked from the mental fields of the people, and there will be none who will be willing to take the quack remedies, but all will look to the Science of Medicine for the relief from suffering and the cure of disease.

To make this organization complete and perpetual, we must rid ourselves of the petty jealousies so common among us and must cultivate with all diligence that greatest of virtues, Love.

"The night has a thousand eyes and the day but one,
Yet the light of a bright world dies with the setting sun.
The mind has a thousand eyes and the heart but one,
But the joy of a whole life dies when love is done."

